# Sex-specific threshold effect of albumin-corrected fructosamine on diabetic retinopathy: a nonlinear relationship in women but linear in men — a cross-sectional study

**DOI:** 10.3389/fendo.2026.1935387

**Published:** 2026-09-16

**Authors:** Qiyun Long, Shuang Kang, Qiyao He, Huaguo Wang, Xing Qi, Sheng Lin, Xuhui Song, Qin Guo, Zejiang Liu

**Affiliations:** Department of Clinical Laboratory Medicine, Ziyang Central Hospital, Ziyang, China

**Keywords:** albumin-corrected fructosamine, diabetic retinopathy, restricted cubic splines, segmented regression, sex differences, threshold effect

## Abstract

**Background:**

Albumin-corrected fructosamine (AlbF) is a short-term glycemic marker that can substitute for HbA1c when HbA1c is unreliable. Studies have linked AlbF to diabetic retinopathy (DR), but it is not known whether the dose-response curve differs between men and women. We investigated this in 1,090 type 2 diabetes patients (433 men, 657 women) from a single Chinese center.

**Methods:**

Restricted cubic splines were used to model the AlbF-DR relationship separately by sex, with threshold identification via segmented logistic regression. Models included age, BMI, diabetes duration, systolic blood pressure, HbA1c, HDL-C, and medications as covariates.

**Results:**

Each 10 μmol/g increase in AlbF raised DR risk in both sexes: men OR 1.83 (95% CI 1.39–2.41), women OR 1.58 (95% CI 1.26–1.99). The RCS curves told a different story. Men followed a linear pattern (P for nonlinearity = 0.751). Women showed a threshold near 62.0 μmol/g (P for nonlinearity < 0.001). Below that value, the OR was 2.57 (95% CI 1.74–3.81); above it, the association was no longer significant (OR 1.29, 95% CI 0.69–2.42; P for interaction < 0.001). Sensitivity analyses consistently reproduced this nonlinear pattern.

**Conclusions:**

The AlbF-DR relationship is linear in men but follows a threshold model in women, with a change point near 62.0 μmol/g. Sex-specific risk modeling may improve DR stratification. Whether the threshold holds in practice will require prospective studies.

## 1 Introduction

Diabetic retinopathy (DR) is a leading cause of preventable blindness in the world (1–3). Glycated hemoglobin A1c (HbA1c), the standard marker for long-term glycemic control, is closely tied to DR risk. However, HbA1c becomes unreliable when conditions alter red blood cell turnover. Anemia, hemoglobinopathies, and chronic kidney disease can all distort HbA1c readings, pushing them above or below true average glycemia (4–6). This has prompted interest in alternative markers. Serum-based markers like fructosamine and glycated albumin capture glycemic control over a shorter window of 2 to 3 weeks and predict microvascular complications roughly as well as HbA1c (7–9). Among them, the albumin-corrected fructosamine index (AlbF) divides fructosamine by serum albumin to account for variations in albumin concentration. Because serum albumin is frequently reduced in chronic disease, malnutrition, and impaired hepatic synthesis, this correction improves the accuracy of the glycemic estimate precisely in the patients in whom HbA1c is least reliable. Several studies have linked AlbF and related fructosamine-based markers to DR (10, 11). Yet the shape of the dose-response curve connecting these markers to DR risk has not been clearly defined, nor has the question of whether this curve might differ by sex. Whether DR risk differs between men and women is debated. Some studies report a higher prevalence in men (12, 13), while others identify female sex as an independent risk factor, particularly in Chinese cohorts (14). The Maastricht Study found no consistent sex differences in how glycemic levels relate to microvascular complications (15). And even if overall prevalence were the same, the shape of the dose-response curve might differ between sexes. That has not been examined directly. The same gap applies to markers like AlbF. Prior work on fructosamine and glycated albumin handled sex only as a confounder to adjust for, not as an effect modifier that could change the association itself. That approach assumes the same model works for both sexes. It has rarely been questioned.

Biologically, there are reasons to look for sex differences. Oxidative stress defense (16, 17), AGE metabolism (18), and estrogen-mediated retinal vascular permeability (19, 20) all differ between men and women. Retinal gene expression shows sex-specific patterns in inflammation regulators (21). None of this proves the AlbF-DR relationship varies by sex, but it makes the question worth asking.

We analyzed 1,090 type 2 diabetes patients from a single center in China using sex-stratified models. Restricted cubic splines (RCS) let us map the AlbF-DR dose-response curves separately for men and women without imposing linearity, and we identified thresholds through nonlinear curve derivative analysis (22, 23). Our hypothesis was that the two curves would not follow the same pattern: women would show a non-monotonic relationship with a threshold, while men would follow a roughly linear trend.

## 2 Methods

### 2.1 Study population

#### 2.1.1 Data source and eligibility criteria

The study cohort was described previously (10). We screened 1,419 patients with type 2 diabetes mellitus (T2DM) who attended the Ophthalmology Department of Ziyang Central Hospital between January 2018 and April 2022. After exclusions (Section 2.1.2), 433 men and 657 women remained for analysis. Patients came from inpatient wards and were identified through electronic medical records.

#### 2.1.2 Inclusion and exclusion criteria

Inclusion and exclusion criteria matched our prior report (10). Briefly, inclusion required a confirmed diagnosis of T2DM. Exclusion criteria comprised: (1) ocular comorbidities (glaucoma, high myopic retinopathy, hypertensive retinopathy, diabetic optic neuropathy); (2) severe systemic conditions (nephrotic syndrome, liver cirrhosis, active malignancy or chemotherapy); and (3) maintenance hemodialysis (n=31). DR was diagnosed as detailed in Section 2.2.2.

### 2.2 Measurements and definitions

#### 2.2.1 Primary exposure: albumin-corrected fructosamine

Serum albumin was measured using the bromocresol green method on an ADVIA 2400 automated clinical chemistry analyzer (Siemens Healthcare Diagnostics, Germany; intra-assay CV 0.88%, inter-assay CV 1.64%). Serum fructosamine was measured using the tetrazolium blue reduction method on the same platform (reagents from Zhongyuan Huiji Biotechnology Co., Ltd., Chongqing, China; intra-assay CV 0.44%, inter-assay CV 2.32%). The fructosamine assay was linear over a range of 0.3–6.0 mmol/L, and the albumin assay was linear over 3.0–60.0 g/L according to the manufacturer’s specifications. Fructosamine values (mmol/L) were converted to μmol/L by multiplying by 1,000. AlbF was calculated as fructosamine (μmol/L) divided by serum albumin (g/L) and is expressed in μmol/g. HbA1c was measured by high-performance liquid chromatography (HPLC). Hemoglobin, lipids, and creatinine were measured using standardized automated methods as previously reported (10).

#### 2.2.2 Outcome: diabetic retinopathy diagnosis

DR was diagnosed by two experienced ophthalmologists based on dilated fundus examination and fundus photography, both blinded to laboratory values. The diagnosis followed the Early Treatment Diabetic Retinopathy Study (ETDRS) criteria. DR was defined as the presence of at least one characteristic retinal lesion in either eye, including microaneurysms, retinal hemorrhages, hard exudates, cotton-wool spots, venous beading, or neovascularization (24). Any discrepancies between the two ophthalmologists were resolved by consensus.

#### 2.2.3 Covariates

Covariates included age, sex, body mass index (BMI), duration of diabetes, systolic blood pressure, HbA1c, high-density lipoprotein cholesterol (HDL-C), smoking status, and menopausal status (in women). Smoking was defined as ever smoker (current or former), obtained from the electronic medical records. Menopausal status was also obtained from medical records. All covariates were measured at baseline on the same day as AlbF measurement. Estimated glomerular filtration rate (eGFR) was calculated using the CKD-EPI 2021 equation as described by Inker et al. (25) and was included as a covariate in sensitivity analyses.

### 2.3 Statistical analysis

#### 2.3.1 Descriptive statistics and missing data handling

Continuous variables are presented as median (interquartile range) and were compared between groups using the Mann–Whitney U test. Categorical variables are expressed as frequencies (percentages) and were compared using the chi-square test or Fisher’s exact test, as appropriate.

The extent and pattern of missing data were similar to those described in our previous report (10). Missingness rates were below 20% for all variables, with no substantial differences between men and women. To improve statistical power and reduce potential bias, multiple imputation by chained equations (MICE) was performed. In the present analysis, 20 imputed datasets were generated [vs. 5 imputations in our prior study (26)] to ensure stable estimates for sex-specific and subgroup analyses. A comparison of key characteristics between the original and imputed datasets showed no substantive differences.

#### 2.3.2 Sex-stratified multivariable logistic regression

To evaluate the association between AlbF and DR separately in men and women, multivariable logistic regression analyses were performed with AlbF entered into the models in two forms: as a continuous variable (per 10 μmol/g increase in AlbF) and as tertiles to assess dose–response relationships. The nested adjustment strategy was identical to that used in our previous publication (10). Briefly, five sequentially adjusted models were constructed:

Model 1: unadjusted.
Model 2: adjusted for age and body mass index (BMI).
Model 3: further adjusted for diabetes duration, systolic blood pressure, and HbA1c.
Model 4: further adjusted for high-density lipoprotein cholesterol (HDL-C) and smoking status (the latter was included only in men, as no women in the cohort were smokers).
Model 5 (primary model): further adjusted for antidiabetic and antihypertensive medications.

For the categorical analyses (tertiles), the lowest category served as the reference, and a test for linear trend was performed by entering the categorical variable as an ordinal term in the model. All results are reported as odds ratios (ORs) with 95% confidence intervals (CIs).

#### 2.3.3 Non-linear dose-response analysis: restricted cubic splines

To formally test whether sex modified the AlbF-DR association, we included a sex × AlbF multiplicative interaction term in the multivariable logistic regression model and assessed its significance. Both models included the full set of covariates (age, BMI, diabetes duration, SBP, HbA1c, HDL-C, antidiabetic and antihypertensive medications, and smoking status in men) and a restricted cubic spline term for AlbF with 3 knots at the 10th, 50th, and 90th percentiles. The null model further included a sex × AlbF linear interaction term to account for potential slope differences, while the full model additionally incorporated interaction terms between sex and the nonlinear spline bases of AlbF. A significant likelihood ratio test indicates that the nonlinear curvature of the AlbF-DR relationship differs between sexes. We additionally evaluated additive interaction between sex and AlbF using the relative excess risk due to interaction (RERI), the attributable proportion due to interaction (AP), and the synergy index (SI), with statistical significance determined by 95% confidence intervals.

#### 2.3.4 Threshold effect analysis (segmented regression) in women

Because no significant nonlinearity was detected in men (P for nonlinearity >0.05), segmented regression was performed only in women. Given the significant nonlinearity observed in women (P for nonlinearity from likelihood ratio test <0.001), segmented logistic regression was applied to estimate the breakpoint where the slope of the association between AlbF and DR changes. The 95% confidence interval of the breakpoint was obtained using the bootstrap percentile method with 1,000 resamples. AlbF values above the 99.5th percentile were trimmed for consistency with the RCS models, excluding 4 women with AlbF > 102.97 μmol/g, yielding n = 653. Odds ratios for AlbF (per 10 μmol/g increase) were calculated separately for the left side (AlbF below the breakpoint) and the right side (AlbF above the breakpoint). The model was adjusted for the same covariates as in the RCS analysis (Model 5). In a sensitivity analysis, the segmented regression was further adjusted for hemoglobin (HGB) to exclude potential confounding by anemia.

#### 2.3.5 Subgroup analysis in women by menopausal status

To explore whether menopausal status modified the threshold effect of AlbF on DR, women were stratified according to their documented menopausal status at the time of hospital admission (premenopausal, n=104; postmenopausal, n=553). Restricted cubic spline (RCS) analyses were repeated within each menopausal subgroup, adjusting for the same covariates as in the primary analysis in women (Model 5). The shape of the dose–response curve, the estimated breakpoint, and the odds ratios on either side of the breakpoint were re-estimated and descriptively compared between the two subgroups. To formally test for effect modification, a likelihood ratio test was performed comparing a model containing the main effects and the spline terms of AlbF with a model that additionally included the product terms between menopausal status and each spline basis. Given the limited sample size of premenopausal women (n=104), results from this subgroup should be interpreted cautiously.

#### 2.3.6 Sensitivity analyses

Several sensitivity analyses were conducted to evaluate the robustness of the findings. First, the segmented regression model in women was re-run with hemoglobin (HGB) added as a continuous covariate. Second, women with serum albumin <35 g/L were excluded to avoid potential bias from severe hypoalbuminemia. Third, a complete-case analysis was performed, repeating all main analyses without any imputation to assess whether missing data influenced the results.

#### 2.3.7 Software and code availability

All statistical analyses were two-tailed, with a significance level set at α = 0.05. The statistical software programs R (version 4.2.2; https://www.r-project.org/) and Free Statistics software (version 2.2) were used to conduct all the analyses (27).

### 2.4 Ethics approval

This study was conducted in accordance with the Declaration of Helsinki and approved by the Ethics Committee of Ziyang Central Hospital (Approval No. 2025-492; dated 12 November 2025). The requirement for informed consent was waived due to the retrospective design and the use of anonymized data.

## 3 Results

### 3.1 Baseline characteristics of the study population

A study flow diagram is presented in **Figure 1**. A total of 1,090 participants were included (433 men, 657 women). Baseline characteristics stratified by sex are shown in **Table 1**. Compared with women, men had significantly lower levels of albumin-corrected fructosamine (AlbF, per 10 μmol/g) (5.0 ± 1.2 vs. 5.3 ± 1.3, P = 0.001), total cholesterol, HDL-C, and LDL-C, but higher eGFR (87.9 ± 26.3 vs. 93.5 ± 21.2 mL/min/1.73m², P<0.001), serum creatinine, uric acid, and hemoglobin (all P<0.001). Men also had higher proportions of ever smokers and ever drinkers (both P<0.001). Diabetes duration was slightly longer in women (median [IQR]: 8.0 [3.0, 10.0] vs. 7.0 [2.0, 10.0] years; P = 0.032). No significant sex differences were observed in age, BMI, or use of antidiabetic or antihypertensive medications. Baseline characteristics of the total population by DR status and sex-stratified by DR status are provided in **Supplementary Tables S1, S2**, respectively.

**Figure 1:**
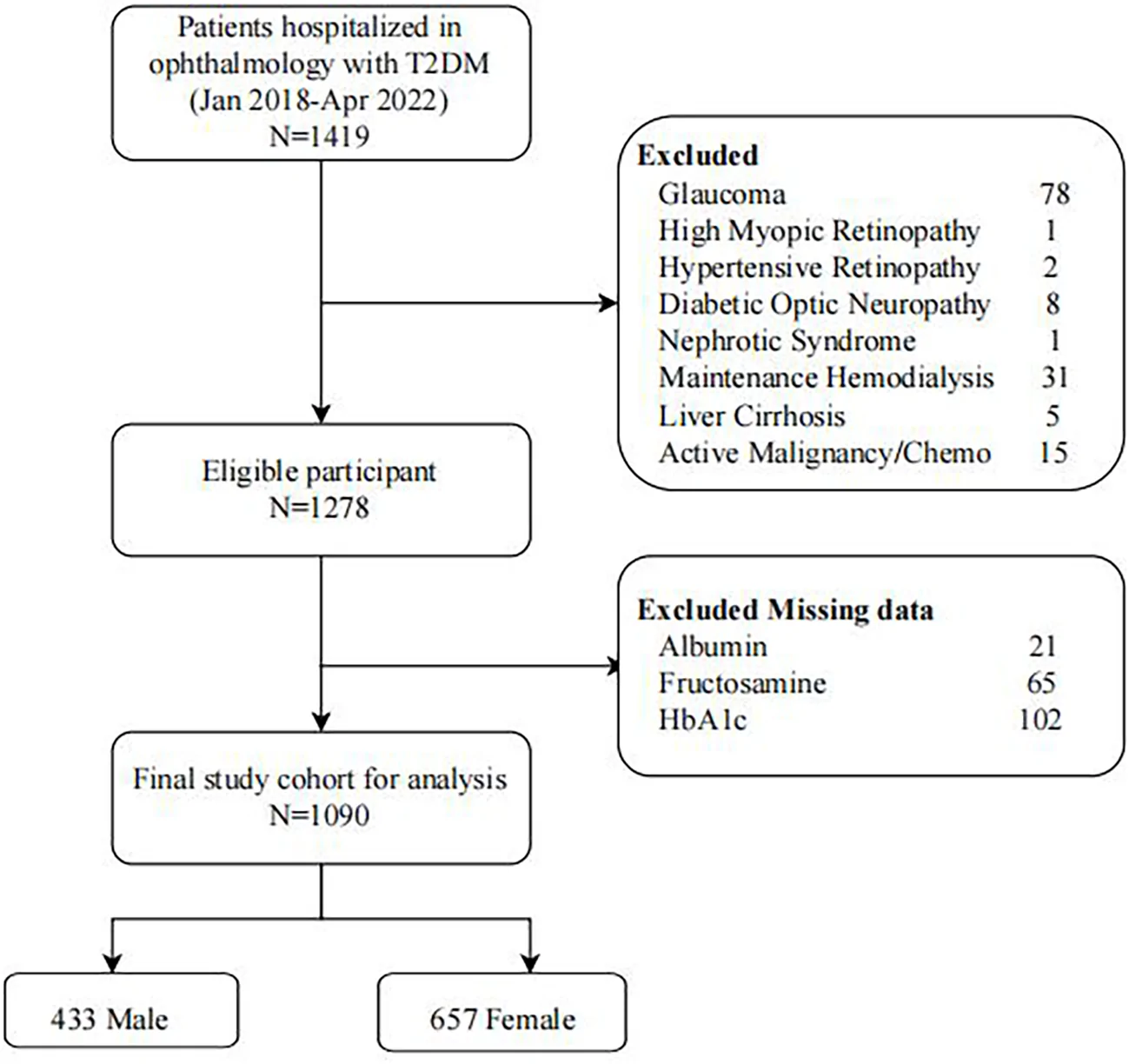
Flowchart of participant selection for the study. This diagram illustrates the sequential process of screening, exclusion, and inclusion of patients with type 2 diabetes mellitus (T2DM) in this retrospective, cross-sectional study.

**Table 1:** Baseline characteristics of the study population stratified by sex.

Variables	Total (n = 1090)	Male (n = 433)	Female (n = 657)	P value
Demographics				
Age, year	63.5 ± 11.0	62.9 ± 12.2	63.9 ± 10.1	0.172
BMI, kg/m²	24.9 ± 3.6	24.6 ± 3.3	25.1 ± 3.7	0.057
SBP, mmHg	137.9 ± 21.1	135.8 ± 20.6	139.2 ± 21.3	0.01
DBP, mmHg	78.9 ± 11.5	79.7 ± 11.9	78.4 ± 11.2	0.062
Smoke, n (%)				< 0.001
Never	995 (91.3)	338 (78.1)	657 (100)	
Ever	95 (8.7)	95 (21.9)	0 (0)	
Alcohol, n (%)				< 0.001
Never	1044 (95.8)	389 (89.8)	655 (99.7)	
Ever	46 (4.2)	44 (10.2)	2 (0.3)	
Occupation, n (%)				< 0.001
Farmer	673 (61.7)	215 (49.7)	458 (69.7)	
Retired	229 (21.0)	129 (29.8)	100 (15.2)	
Other1	188 (17.2)	89 (20.6)	99 (15.1)	
Laboratory measurements				
AlbF, per 10 μmol/g*	5.2 ± 1.3	5.0 ± 1.2	5.3 ± 1.3	0.001
glucose, mmol/L	8.6 ± 4.2	8.4 ± 4.1	8.7 ± 4.2	0.207
Fructosamine, mmol/L	2.1 ± 0.5	2.0 ± 0.5	2.1 ± 0.5	0.001
HbA1c, %(mmol/mol)	8.5 ± 2.1	8.3 ± 2.0	8.6 ± 2.2	0.039
Total cholesterol, mmol/L	5.1 ± 1.2	4.8 ± 1.1	5.3 ± 1.3	< 0.001
Triglycerides, mmol/L	1.5 (1.1, 2.2)	1.5 (1.0, 2.2)	1.5 (1.2, 2.3)	0.093
HDL-C, mmol/L	1.5 ± 0.4	1.4 ± 0.4	1.6 ± 0.4	< 0.001
LDL-C, mmol/L	2.8 ± 0.9	2.7 ± 0.9	2.9 ± 1.0	0.002
Urea, mmol/L	6.6 ± 2.8	7.1 ± 3.2	6.2 ± 2.5	< 0.001
Creatinine, μmol/L	61.0 (48.0, 79.0)	74.0 (63.0, 97.0)	52.0 (44.0, 66.0)	< 0.001
Uric acid, μmol/L	336.4 ± 101.3	376.7 ± 105.5	309.8 ± 89.0	< 0.001
Total protein, g/L	67.1 ± 6.6	66.4 ± 6.8	67.5 ± 6.4	0.006
Albumin, g/L	40.8 ± 4.9	40.8 ± 5.2	40.7 ± 4.8	0.768
eGFR, mL/min/1.73m²	91.3 ± 23.5	87.9 ± 26.3	93.5 ± 21.2	< 0.001
Pre-albumin, g/L	237.3 ± 51.3	251.8 ± 53.1	227.8 ± 47.8	< 0.001
White blood cell, 10^9^/L	6.3 ± 1.8	6.4 ± 1.9	6.3 ± 1.8	0.362
Neutrophil, 10^9^/L	3.9 ± 1.6	4.0 ± 1.7	3.8 ± 1.5	0.04
Lymphocyte, 10^9^/L	1.8 ± 0.6	1.7 ± 0.6	1.9 ± 0.6	< 0.001
Red blood cell, 10^12^/L	4.3 ± 0.6	4.5 ± 0.7	4.2 ± 0.5	< 0.001
Hemoglobin, g/L	129.0 ± 18.8	136.8 ± 19.6	123.8 ± 16.3	< 0.001
Platelet, 10^9^/L	182.3 ± 63.7	172.5 ± 59.1	188.7 ± 65.9	< 0.001
Comorbidities & medications				
DR, n (%)	459 (42.1)	167 (38.6)	292 (44.4)	0.055
DM duration, years	8.0 (2.0, 10.0)	7.0 (2.0, 10.0)	8.0 (3.0, 10.0)	0.032
Hypertension, n (%)	565 (51.8)	226 (52.2)	339 (51.6)	0.847
DM meds, n (%)	783 (71.8)	305 (70.4)	478 (72.8)	0.405
HTN meds, n (%)	386 (35.4)	152 (35.1)	234 (35.6)	0.863
Menopause (women only), n (%)	Not applicable	Not applicable	553 (84.2)	Not applicable

^1^ The “Other” category included all participants who were not farmers or retirees, encompassing both employed individuals across various sectors and those unemployed. *AlbF was entered as a continuous variable scaled per 10 μmol/g to facilitate interpretation.

DR, diabetic retinopathy; BMI, Body Mass Index; DBP, Diastolic blood pressure; SBP, Systolic pressure; AlbF, Fructosamine/Albumin; HDL-C, High density lipoprotein cholesterol; LDL-C, Low-Density Lipoprotein Cholesterol; eGFR, estimated glomerular filtration rate; DM, diabetes mellitus; DM meds/HTN meds, use of glucose-lowering or antihypertensive medication; HbA1c, Glycated hemoglobin.

### 3.2 Association between AlbF and DR in men and women

Multivariable logistic regression analyses were performed separately in men and women with AlbF entered as a continuous variable (per 10 μmol/g increase). The fully adjusted model (Model 5) included age, BMI, diabetes duration, systolic blood pressure, HbA1c, HDL-C, antidiabetic medications, and antihypertensive medications; smoking status (never/ever) was additionally adjusted in men (no female participants reported smoking history). **Table 2** shows the continuous-variable results.

**Table 2:** Association between AlbF (continuous, per 10 μmol/g) and diabetic retinopathy in men and women (Model 5).

Sex	Adjusted OR (95% CI)	P value
Men (n=433)	1.83 (1.39–2.41)	<0.001
Women (n=657)	1.58 (1.26–1.99)	<0.001

Model 5 adjusted for age, BMI, diabetes duration, SBP, HbA1c, HDL-C, antidiabetic medications, and antihypertensive medications. In men only, smoking status was additionally adjusted (no women smoked).

BMI, Body Mass Index; SBP, Systolic pressure; AlbF, Fructosamine/Albumin; HDL-C, High density lipoprotein cholesterol; HbA1c, Glycated hemoglobin.

In men, each 10 μmol/g increase in AlbF was associated with a significantly increased risk of DR (adjusted OR = 1.83, 95% CI: 1.39–2.41, P < 0.001). In women, the corresponding OR was 1.58 (95% CI: 1.25–1.98, P < 0.001). When AlbF was analyzed in tertiles (Model 5), both sexes showed progressively higher DR risk across increasing tertiles (both P for trend < 0.001; **Supplementary Table S3**). The associations were consistent across all adjustment models (**Supplementary Table S4**).

### 3.3 Sex specific dose–response relationship: restricted cubic splines

To model the dose–response relationship between AlbF and DR, restricted cubic spline (RCS) curves with 3 knots were fitted separately by sex. Adjusted covariates mirrored Model 5 (age, BMI, diabetes duration, SBP, HbA1c, HDL-C, antidiabetic and antihypertensive medications), with additional adjustment for smoking status (never/ever) in men (**Figure 2**).

**Figure 2:**
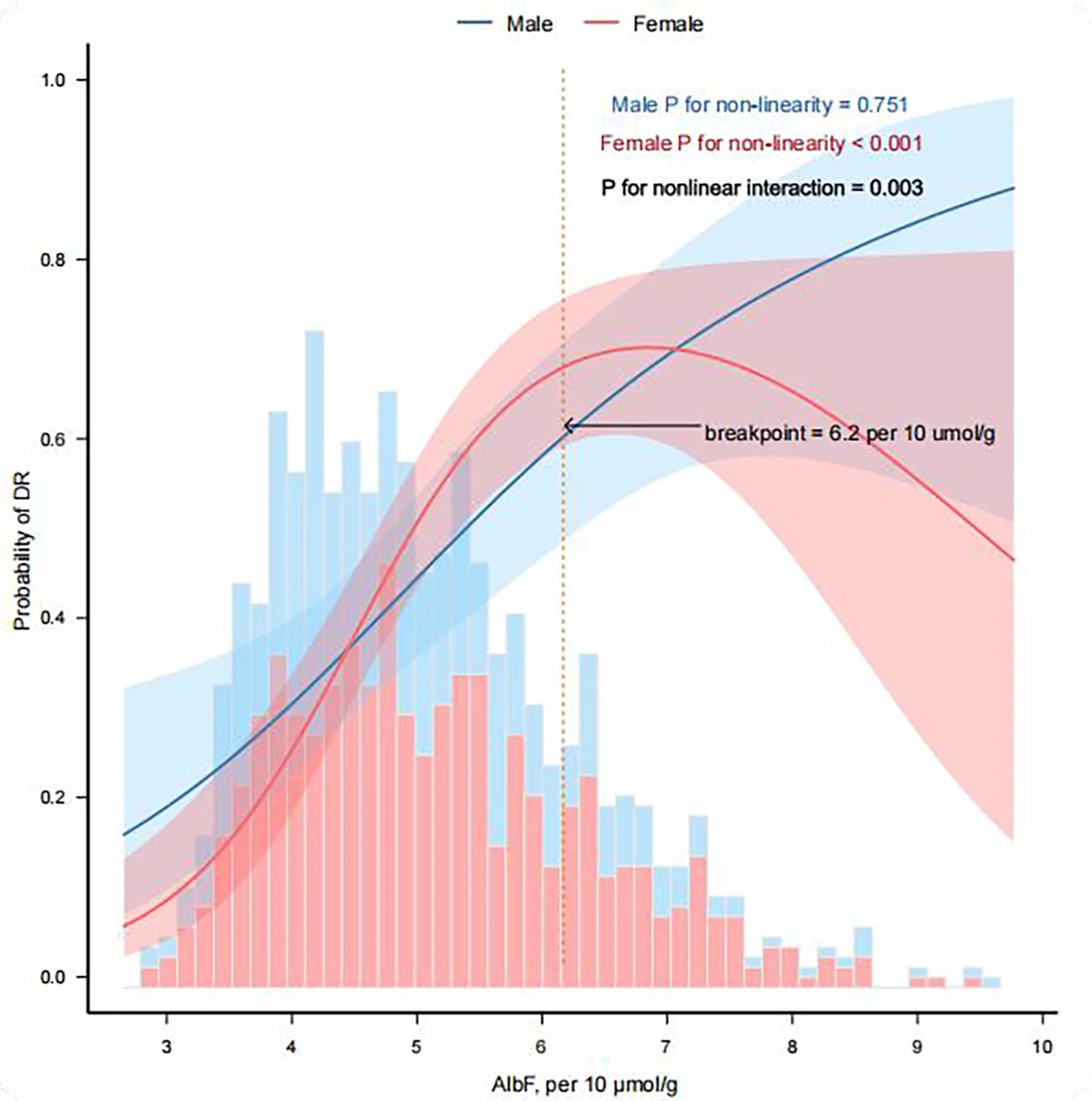
Sex-specific dose–response relationship between albumin-corrected fructosamine (AlbF) and diabetic retinopathy (DR). Restricted cubic spline (RCS) curves with 3 knots (placed at the 10th, 50th, and 90th percentiles) were fitted separately for male (blue) and female (red). The models were adjusted for age, body mass index, diabetes duration, systolic blood pressure, HbA1c, HDL cholesterol, antidiabetic and antihypertensive medications; smoking was additionally adjusted in men (no women smoked). Shaded areas show 95% confidence bands. In men, AlbF is associated with DR in an approximately linear, positive manner (P for nonlinearity = 0.751), indicating a steady rise in risk with higher AlbF. In women, a clear saturation pattern (plateau effect) is observed: the risk of DR increases steeply below the breakpoint (AlbF < 62.0 μmol/g) and then flattens above it. The breakpoint is indicated by a vertical dashed line at AlbF = 62.0 μmol/g. Nonlinearity in women is statistically significant (P < 0.001). The nonlinear curvature of the dose–response relationship differed significantly between sexes (P for nonlinear interaction = 0.003).

In men, the RCS curve showed a nearly linear positive association (P for nonlinearity = 0.751 by likelihood ratio test). Women had a threshold pattern with significant nonlinearity (P for nonlinearity < 0.001), suggesting distinct dose-response architectures between sexes. A formal comparison of nested models confirmed that the nonlinear curvature differed significantly between men and women: a model additionally including sex × spline-basis interaction terms fit better than a model allowing only a linear sex × AlbF interaction (nonlinear interaction: Wald χ² = 8.90, df = 1, P = 0.003; likelihood ratio χ² = 8.85, df = 1, P = 0.003), whereas the linear sex × AlbF interaction was not significant (P = 0.447). On the multiplicative scale, the sex × AlbF interaction was not significant (OR = 1.41, 95% CI: 0.76–2.61; P = 0.28). On the additive scale, none of the measures reached statistical significance by their 95% CIs (RERI = 0.65, 95% CI: −0.40 to 1.70; AP = 0.25, 95% CI: −0.12 to 0.63; SI = 1.71, 95% CI: 0.60 to 4.85).

### 3.4 Threshold effect of AlbF on DR in women

Because the nonlinear association in women was significant (P for nonlinearity < 0.001), we performed segmented logistic regression (adjusted for the same covariates as Model 5) to identify a potential breakpoint. The analysis identified a breakpoint at 62.0 μmol/g (95% CI: 61.27–62.72). Because data-driven breakpoints obtained from segmented regression and restricted cubic splines are sensitive to sample composition and modeling choices, this value should be interpreted cautiously as a hypothesis-generating estimate; the robust finding is the sex-specific nonlinearity itself rather than the precise cut-off. Below this threshold, each 10 μmol/g increase in AlbF was significantly associated with an elevated risk of DR (adjusted OR = 2.57, 95% CI: 1.74–3.81; P < 0.001). Above the threshold, the association was attenuated and no longer statistically significant (adjusted OR = 1.29, 95% CI: 0.69–2.42; P = 0.429). A likelihood ratio test confirmed the segmented model fit better than a simple linear model (P = 0.011; **Table 3**).

**Table 3:** Segmented regression analysis of the threshold effect of AlbF on diabetic retinopathy in women.

Parameter	Estimate (95% CI)	P value
Breakpoint		
AlbF (μmol/g)	62.0 (61.27–62.72)	NA
Odds ratio per 10 μmol/g increase		
Below breakpoint (n = 510)	2.57 (1.74–3.81)	<0.001
Above breakpoint (n = 143)	1.29 (0.69–2.42)	0.429
Nonlinearity tests		
Likelihood ratio test (segmented vs. linear)	–	0.011

Model adjusted for age, BMI, diabetes duration, SBP, HbA1c, HDL-C, antidiabetic medications, and antihypertensive medications. AlbF values above the 99.5th percentile were trimmed for consistency with the RCS models, excluding 4 women with AlbF > 102.97 μmol/g, yielding n = 653.

BMI, Body Mass Index; SBP, Systolic pressure; AlbF, Fructosamine/Albumin; HDL-C, High density lipoprotein cholesterol; HbA1c, Glycated hemoglobin.

To validate this threshold, we stratified women into two groups by the estimated breakpoint (AlbF <62.0 μmol/g vs. ≥62.0 μmol/g) and tested for interaction. The interaction term between AlbF (per 10 μmol/g) and the threshold group was significant (P for interaction < 0.001), confirming that the AlbF-DR association differs on either side of the breakpoint. Sensitivity analyses further examined this threshold effect (Section 3.6; **Table 4**).

**Table 4:** Sensitivity analyses for the association between AlbF (per 10 μmol/g) and diabetic retinopathy in women under different adjustment conditions.

Sensitivity analysis	n.total	n.event (%)	OR (95%CI)	P value
+ adjustment for HGB	657	292 (44.4)	1.39 (1.09–1.76)	0.008
+ adjustment for eGFR	657	292 (44.4)	1.46 (1.15–1.86)	0.002
Excluding albumin <35 g/L	585	234 (40)	1.57 (1.20–2.05)	0.001
Complete-case without imputation	517	219 (42.4)	1.72 (1.30–2.28)	<0.001

Model adjusted for age, BMI, diabetes duration, SBP, HbA1c, HDL-C, antidiabetic medications, and antihypertensive medications.

BMI, Body Mass Index; SBP, Systolic pressure; AlbF, Fructosamine/Albumin; HDL-C, High density lipoprotein cholesterol; HbA1c, Glycated hemoglobin.

### 3.5 Subgroup analysis by menopausal status in women

To assess whether menopausal status modified the threshold effect of AlbF on DR, we stratified women into premenopausal (n=104) and postmenopausal (n=553) subgroups. Within each stratum, multivariable logistic regression (Model 5) and restricted cubic spline (RCS) analyses were performed. Visual inspection of the RCS curves suggested similar nonlinear patterns in both subgroups, but formal segmented regression to estimate a breakpoint was not conducted due to the limited sample size in the premenopausal group.

In premenopausal women, AlbF (per 10 μmol/g) was statistically significantly associated with DR (adjusted OR = 1.87, 95% CI: 1.08–3.23; P = 0.025). In postmenopausal women, the association was also significant (adjusted OR = 1.52, 95% CI: 1.17–1.98; P = 0.002), and RCS analysis confirmed a significant nonlinear relationship (P for nonlinearity < 0.001).

A formal test for interaction between menopausal status (pre- vs. postmenopausal) and continuous AlbF was not statistically significant (P for interaction = 0.931). Menopausal status did not substantially alter the overall AlbF-DR relationship, though the premenopausal analysis was limited by sample size and statistical power (**Supplementary Table S5**; **Supplementary Figure S2**).

### 3.6 Sensitivity analyses

We ran several sensitivity analyses to test whether the primary association and threshold effect held under different analytical choices (**Table 4**; **Supplementary Table S6**).

Adjusting for hemoglobin (HGB, continuous) left the positive association intact (adjusted OR = 1.39, 95% CI: 1.09–1.76; P = 0.008), and RCS analysis still showed significant nonlinearity (P for nonlinearity < 0.001; breakpoint 51.60 μmol/g, **Supplementary Figure S1A**). Excluding women with serum albumin <35 g/L (n=72) gave similar results (adjusted OR = 1.57, 95% CI: 1.20–2.05; P = 0.001; RCS P for nonlinearity < 0.001, breakpoint 51.60 μmol/g, **Supplementary Figure S1B**). Complete-case analysis without imputation (n=517) confirmed the direction and significance of the association (adjusted OR = 1.72, 95% CI: 1.30–2.28; P < 0.001). Additionally adjusting for eGFR had no material effect on the estimate (adjusted OR = 1.46, 95% CI: 1.15–1.86; P = 0.002).

The breakpoint from the sensitivity RCS analyses (51.60 μmol/g) was lower than the primary segmented regression estimate (62.0 μmol/g). This is expected: segmented regression directly estimates the point of slope change, whereas RCS breakpoints were identified by curve inspection. Excluding hypoalbuminemic patients and adjusting for HGB also shifted the data distribution, which can affect where different algorithms place the point of maximal slope change. The nonlinear pattern was significant across all scenarios (P for nonlinearity < 0.001), confirming it is not an artifact of any single analytical choice.

## 4 Discussion

Our study found a sex difference in the AlbF-DR relationship: linear in men, threshold in women. In sex-stratified multivariable analyses, each 10 μmol/g increment in AlbF was associated with a 1.83-fold (95% CI: 1.39–2.41) and 1.58-fold (95% CI: 1.26–1.99) increased odds of DR in men and women, respectively. Restricted cubic spline analyses showed that the AlbF-DR relationship was essentially linear in men (P for nonlinearity = 0.751) but nonlinear in women (P for nonlinearity < 0.001). Segmented logistic regression identified a breakpoint at 62.0 μmol/g in women (likelihood ratio test for segmented vs. linear model: P = 0.011). Stratifying women by this breakpoint confirmed the threshold: AlbF was associated with DR below 62.0 μmol/g (n = 510, P < 0.001) but not above it (n = 143, P = 0.429). A formal nonlinear interaction test confirmed that the curvature of the dose–response relationship differed significantly between sexes (Wald χ² = 8.90, P = 0.003), whereas neither multiplicative (P = 0.28) nor additive interaction measures were significant, indicating that the sex difference resides in the shape of the dose–response curve rather than in the average effect size. Because the study is cross-sectional, these associations do not establish causality or temporality and should be interpreted accordingly.

Several mechanisms could explain the threshold effect in women. The nonlinearity may reflect saturation of AGE formation or receptor binding kinetics. Once albumin glycation exceeds a stoichiometric ceiling, further modification may not proportionally increase RAGE activation or downstream inflammatory signaling (28–30). Women have greater endogenous antioxidant capacity than men, partly through estrogen-mediated upregulation of glutathione and superoxide dismutase (16, 17). This redox buffering could neutralize glycation-induced oxidative damage up to a critical load, beyond which compensatory mechanisms are overwhelmed. Estrogen also modulates vascular permeability by regulating tight junction proteins and endothelial nitric oxide synthase, and could alter the retinal microvascular response to AlbF (19, 20, 31). The absence of effect modification by menopausal status (P for interaction = 0.931) argues against a direct estrogen-dependent mechanism, though this null finding should be interpreted cautiously given the small premenopausal subgroup (n = 104). The linear association in men fits a cumulative damage model where each increase in AlbF proportionally raises DR risk without a saturable protective mechanism.

The sex-specific pattern we identified is consistent with a growing body of evidence documenting sexual dimorphism in glycation biology and diabetic complications (32, 33). Some studies suggest that women with type 2 diabetes show a stronger association between glycemic variability and microvascular complications compared with men (15, 34). Our findings extend this literature by demonstrating that the dose-response architecture itself, linear in men versus threshold in women, differs between sexes. The breakpoint estimate of 62.0 μmol/g provides a candidate cut-point for risk stratification, though this threshold requires independent validation before clinical use. Sensitivity analyses that additionally adjusted for hemoglobin or excluded women with serum albumin < 35 g/L confirmed that the nonlinear pattern remained significant across all scenarios (P for nonlinearity < 0.001 in every model). The dose-response plateau above the breakpoint is worth noting: among women above 62.0 μmol/g, further AlbF elevation conferred no additional DR risk. This pattern challenges the conventional assumption of a monotonic glycemic-DR relationship in all patients (35) and supports sex-specific risk modeling (33).

These findings carry potential clinical implications. If validated in prospective cohorts, the 62.0 μmol/g threshold may represent a candidate sex-specific marker for DR risk stratification, pending prospective validation in women with type 2 diabetes. Women with AlbF levels below this threshold, in whom the risk slope is steepest, might be strong candidates for closer monitoring and early ophthalmologic referral, whereas the marginal benefit of further glycemic reduction may be smaller in those already above the threshold. These findings illustrate how risk stratification could eventually be tailored by sex, moving toward more personalized diabetes care (36). Our results also suggest that the AlbF index may complement HbA1c in capturing short-term glycemic exposure relevant to DR pathogenesis (7, 10), particularly when HbA1c is unreliable (e.g., in anemia or hemoglobinopathies) (4). As a 2–3 week integrated measure of glycemia, AlbF reflects the glycemic window most relevant to DR pathogenesis, and it can be measured from the same serum sample as routine biochemistry on automated analyzers at no additional cost. However, the cross-sectional design precludes causal inference, and the breakpoint threshold requires validation in longitudinal cohorts before clinical application.

The cross-sectional design limits our ability to determine whether the sex-specific threshold reflects a stable biological phenomenon or emerges from temporal or survival biases inherent to single-occasion sampling. Longitudinal studies tracking within-individual AlbF trajectories and DR onset are needed to confirm that the nonlinear pattern in women is reproducible over time. Participants were recruited from a single center, which may limit generalizability to other populations or healthcare settings. Despite adjustment for multiple covariates, residual confounding from unmeasured factors such as dietary antioxidant intake, physical activity, and socioeconomic status cannot be excluded, and these factors may differ between sexes in ways that influence the shape of the AlbF-DR relationship. Additional unmeasured factors — including detailed hepatic function, menopausal status effects, and obesity measures beyond BMI — could likewise contribute to the observed sex difference and cannot be fully excluded. The premenopausal subgroup (n = 104) was small, limiting statistical power to detect effect modification by menopausal status. The AlbF breakpoint was derived from cross-sectional, hypothesis-generating analyses and requires independent confirmation in external cohorts.

Strengths of the study include sex-stratified modeling with separate analyses for men and women, formal testing of nonlinearity using restricted cubic splines, breakpoint identification via segmented regression with objective optimization, threshold validation through stratified subgroup analysis, and sensitivity analyses that confirmed the nonlinear pattern across different model specifications and covariate adjustments.

## 5 Conclusions

This study found a sex difference in the dose-response relationship between AlbF and DR: linear in men, threshold in women with a breakpoint at approximately 62.0 μmol/g. The saturable pattern in women suggests that AlbF contributes to DR pathogenesis through sex-specific biological pathways that may involve differential AGE metabolism, oxidative stress buffering, or vascular permeability regulation. These results support sex-stratified analyses in diabetes complications research and provide a candidate threshold for future investigation. Prospective cohort studies and mechanistic experiments are needed to validate this sex-specific breakpoint and clarify the molecular basis of sexual dimorphism in AlbF-mediated retinal injury.

## Data Availability

The data analyzed in this study is subject to the following licenses/restrictions: The datasets used and/or analyzed during the current study are available from the corresponding author on reasonable request. Requests to access these datasets should be directed to Qiyun Long, zhoulp2@126.com.
