# Supplementary material for: Sex-specific threshold effect of albumin-corrected fructosamine on diabetic retinopathy: a nonlinear relationship in women but linear in men — a cross-sectional study
Source: Front Endocrinol (Lausanne). 2026 Sep 16;17:1935387. doi: 10.3389/fendo.2026.1935387 (PMC13623703; doi:10.3389/fendo.2026.1935387)
Supplement: Supplementary file 1 [file SupplementaryFile1.pdf]

Supplementary Material Files

To: Sex-specific threshold effect of albumin-corrected fructosamine on diabetic retinopathy: a nonlinear relationship in women but linear in men

Supplementary Table 1. Demographic and Clinical Characteristics of Participants With and Without Diabetic Retinopathy

Supplementary Table 2. Baseline characteristics of men and women stratified by diabetic retinopathy (DR) status

Supplementary Table 3. Tertile analysis of AlbF with DR in men and women (Model 5)

Supplementary Table 4. Stepwise logistic regression results for AlbF (continuous, per 10 μmol/g) and diabetic retinopathy in men and women

Supplementary Table 5. Association between AlbF (per 10 μmol/g) and diabetic retinopathy in women, stratified by menopausal status

Supplementary Table 6. Association between AlbF and diabetic retinopathy in women using complete-case data without imputation (n = 517, Model 5)

Supplementary Figure 1. Sex-specific threshold effect of albumin-corrected fructosamine (AlbF) on diabetic retinopathy (DR): sensitivity analyses in women.

Supplementary Figure 2. Restricted cubic spline curves for the association between AlbF and diabetic retinopathy in women, stratified by menopausal status.

Table S1. Demographic and Clinical Characteristics of Participants With and Without Diabetic Retinopathy

Variables	Total (n = 1090)	NDR (n = 631)	DR (n = 459)	p value
**Demographics**				
Age, year	63.5 ± 11.0	66.9 ± 10.4	58.7 ± 10.0	< 0.001
Sex, n (%)				0.055
Male	433 (39.7)	266 (42.2)	167 (36.4)	
Female	657 (60.3)	365 (57.8)	292 (63.6)	
BMI, kg/m²	24.9 ± 3.6	25.2 ± 3.7	24.5 ± 3.4	0.003
SBP, mmHg	137.9 ± 21.1	137.4 ± 19.8	138.5 ± 22.7	0.38
DBP, mmHg	78.9 ± 11.5	78.8 ± 10.3	79.1 ± 12.9	0.73
Smoke, n (%)				0.513
Never	995 (91.3)	573 (90.8)	422 (91.9)	
Ever	95 (8.7)	58 (9.2)	37 (8.1)	
Alcohol, n (%)				0.025
Never	1044 (95.8)	597 (94.6)	447 (97.4)	
Ever	46 (4.2)	34 (5.4)	12 (2.6)	
Occupation, n (%)				< 0.001
Farmer	673 (61.7)	360 (57.1)	313 (68.2)	
Retired	229 (21.0)	165 (26.1)	64 (13.9)	
Other[1]	188 (17.2)	106 (16.8)	82 (17.9)	
**Laboratory measurements**				
AlbF, per 10 µmol/g*	5.2 ± 1.3	4.9 ± 1.2	5.5 ± 1.3	< 0.001
glucose, mmol/L	8.6 ± 4.2	8.7 ± 4.2	8.5 ± 4.1	0.463
Fructosamine, mmol/L	2.1 ± 0.5	2.0 ± 0.5	2.1 ± 0.4	< 0.001
HbA1c, %(mmol/mol)	8.5 ± 2.1	8.3 ± 2.1	8.8 ± 2.1	< 0.001
Total cholesterol, mmol/L	5.1 ± 1.2	5.0 ± 1.1	5.3 ± 1.3	< 0.001
Triglycerides, mmol/L	1.5 (1.1, 2.2)	1.6 (1.1, 2.3)	1.4 (1.0, 2.2)	0.057
HDL-C, mmol/L	1.5 ± 0.4	1.5 ± 0.4	1.6 ± 0.5	< 0.001
LDL-C, mmol/L	2.8 ± 0.9	2.8 ± 0.9	2.9 ± 1.0	0.244
Urea, mmol/L	6.6 ± 2.8	6.2 ± 2.4	7.1 ± 3.2	< 0.001
Creatinine, µmol/L	61.0 (48.0, 79.0)	59.0 (48.0, 75.0)	64.0 (48.5, 87.0)	< 0.001
Uric acid, µmol/L	336.4 ± 101.3	328.1 ± 103.2	347.8 ± 97.5	0.002
eGFR, mL/min/1.73m²	91.3 ± 23.5	92.6 ± 20.7	89.4 ± 26.7	0.028
Total protein, g/L	67.1 ± 6.6	68.0 ± 6.1	65.7 ± 7.0	< 0.001
Albumin, g/L	40.8 ± 4.9	41.9 ± 4.2	39.2 ± 5.4	< 0.001
Pre-albumin, g/L	237.3 ± 51.3	237.8 ± 50.8	236.7 ± 52.1	0.712
White blood cell, 10^9/L	6.3 ± 1.8	6.4 ± 1.9	6.2 ± 1.7	0.024
Neutrophil, 10^9/L	3.9 ± 1.6	4.0 ± 1.7	3.8 ± 1.5	0.037
Lymphocyte, 10^9/L	1.8 ± 0.6	1.8 ± 0.6	1.8 ± 0.6	0.338
Red blood cell, 10^{12}/L	4.3 ± 0.6	4.4 ± 0.6	4.1 ± 0.6	< 0.001
Hemoglobin, g/L	129.0 ± 18.8	133.6 ± 17.4	122.6 ± 18.7	< 0.001
Platelet, 10^9/L	182.3 ± 63.7	182.1 ± 67.6	182.5 ± 58.1	0.918
**Comorbidities & medications**				

DM duration, years	8.0 (2.0, 10.0)	4.0 (1.0, 10.0)	10.0 (6.0, 14.0)	< 0.001
Hypertension, n (%)				0.223
No	525 (48.2)	294 (46.6)	231 (50.3)	
Yes	565 (51.8)	337 (53.4)	228 (49.7)	
DM meds, n (%)				< 0.001
No	307 (28.2)	265 (42)	42 (9.2)	
Yes	783 (71.8)	366 (58)	417 (90.8)	
HTN meds, n (%)				0.477
No	704 (64.6)	402 (63.7)	302 (65.8)	
Yes	386 (35.4)	229 (36.3)	157 (34.2)	
Menopause, n (%)				< 0.001
No	104 (15.8)	42 (11.5)	62 (21.2)	
Yes	553 (84.2)	323 (88.5)	230 (78.8)	

Data are presented as mean (standard deviation), median (interquartile range), or number (percentage). Multiple imputation was used to handle missing values for baseline characteristics.

[1]The “Other” category included all participants who were not farmers or retirees, encompassing both employed individuals across various sectors and those unemployed.

Abbreviations: NDR, non-diabetic retinopathy; DR, diabetic retinopathy; BMI, Body Mass Index; DBP, Diastolic blood pressure; SBP, Systolic pressure; HbA1c, Glycated hemoglobin; AlbF, Fructosamine/Albumin; HDL-C, High density lipoprotein cholesterol; LDL-C, Low-Density Lipoprotein Cholesterol; eGFR, estimated glomerular filtration rate; DM, diabetes mellitus; DM meds/HTN meds, use of glucose-lowering or antihypertensive medication.

Table S2. Baseline characteristics of men and women stratified by diabetic retinopathy (DR) status

Variables	Male (n=433)			Female (n=657)		
	NDR (n=266)	DR (n=167)	P value	NDR (n=365)	DR (n=292)	P value
**Demographics**						
Age, years	66.1 ± 11.5	57.9 ± 11.6	<0.001	67.6 ± 9.4	59.2 ± 9.0	<0.001
BMI, kg/m²	25.1 ± 3.3	23.9 ± 3.2	<0.001	25.2 ± 3.9	24.8 ± 3.4	0.188
SBP, mmHg	134.8 ± 19.7	137.4 ± 22.0	0.206	139.2 ± 19.8	139.1 ± 23.1	0.959
DBP, mmHg	79.2 ± 10.6	80.6 ± 13.7	0.215	78.6 ± 10.1	78.2 ± 12.4	0.651
Smoke, n (%)			0.932			–
Never	208 (78.2)	130 (77.8)		365 (100)	292 (100)	
Ever	58 (21.8)	37 (22.2)		0 (0)	0 (0)	
Alcohol, n (%)			0.051			1
Never	233 (87.6)	156 (93.4)		364 (99.7)	291 (99.7)	
Ever	33 (12.4)	11 (6.6)		1 (0.3)	1 (0.3)	
Occupation, n (%)			<0.001			0.024
Farmer	117 (44.0)	98 (58.7)		243 (66.6)	215 (73.6)	
Retired	97 (36.5)	32 (19.2)		68 (18.6)	32 (11.0)	
Other[1]	52 (19.5)	37 (22.2)		54 (14.8)	45 (15.4)	
**Laboratory variables**						
AlbF.10 (per 10 µmol/g)	4.8 ± 1.2	5.3 ± 1.2	<0.001	5.0 ± 1.3	5.7 ± 1.3	<0.001
AlbF, µmol/g	48.4 ± 11.6	53.0 ± 12.1	<0.001	49.5 ± 12.9	56.9 ± 12.7	<0.001
glucose, mmol/L	8.8 ± 4.3	7.8 ± 3.8	0.022	8.6 ± 4.1	8.9 ± 4.2	0.438
Fructosamine, mmol/L	2.0 ± 0.5	2.0 ± 0.4	0.561	2.1 ± 0.5	2.2 ± 0.4	<0.001
Glycated hemoglobin, %(mmol/mol)	8.2 ± 2.0	8.5 ± 2.0	0.243	8.3 ± 2.2	9.0 ± 2.1	<0.001
Total cholesterol, mmol/L	4.7 ± 1.1	5.0 ± 1.2	0.016	5.2 ± 1.2	5.4 ± 1.4	0.02

Triglycerides, mmol/L	1.6 (1.1–2.3)	1.3 (0.9–2.0)	<0.001	1.5 (1.2–2.2)	1.6 (1.1–2.4)	0.706
HDL-C, mmol/L	1.4 ± 0.4	1.5 ± 0.5	0.014	1.6 ± 0.4	1.6 ± 0.4	0.032
LDL-C, mmol/L	2.7 ± 0.9	2.7 ± 0.9	0.542	2.9 ± 0.9	2.9 ± 1.0	0.434
Urea, mmol/L	6.6 ± 2.7	7.8 ± 3.7	<0.001	5.9 ± 2.1	6.7 ± 2.8	<0.001
Creatinine, µmol/L	71.0 (62.0–89.0)	81.0 (65.0–113.0)	<0.001	51.0 (43.0–61.0)	55.0 (45.0–73.7)	<0.001
Uric acid, µmol/L	369.7 ± 108.8	387.9 ± 99.2	0.082	297.8 ± 87.3	324.9 ± 89.0	<0.001
eGFR, mL/min/1.73m²	90.2 ± 23.6	84.2 ± 29.7	0.02	94.3 ± 18.2	92.4 ± 24.4	0.254
Total protein, g/L	67.3 ± 6.3	64.9 ± 7.3	<0.001	68.5 ± 5.9	66.2 ± 6.8	<0.001
Albumin, g/L	41.9 ± 4.4	39.1 ± 6.0	<0.001	41.9 ± 4.2	39.2 ± 5.0	<0.001
Pre-albumin, g/L	251.9 ± 51.2	251.6 ± 56.3	0.962	227.6 ± 48.1	228.1 ± 47.5	0.89
White blood cell, 10^9/L	6.5 ± 1.9	6.2 ± 1.9	0.091	6.4 ± 1.8	6.2 ± 1.6	0.147
Neutrophil, 10^9/L	4.1 ± 1.8	3.9 ± 1.6	0.283	3.9 ± 1.6	3.7 ± 1.4	0.092
Lymphocyte, 10^9/L	1.7 ± 0.6	1.6 ± 0.5	0.024	1.9 ± 0.6	1.9 ± 0.6	0.847
Red blood cell, 10^{12}/L	4.6 ± 0.6	4.3 ± 0.7	<0.001	4.3 ± 0.5	4.0 ± 0.5	<0.001
Hemoglobin, g/L	141.7 ± 16.9	129.1 ± 21.1	<0.001	127.7 ± 15.4	118.9 ± 16.1	<0.001
Platelet, 10^9/L	170.8 ± 63.2	175.3 ± 51.8	0.446	190.3 ± 69.5	186.7 ± 61.1	0.476
**Comorbidities & medications**						
DM duration, years	4.0 (1.0–10.0)	10.0 (5.0–14.0)	<0.001	5.0 (1.0–10.0)	10.0 (7.8–14.0)	<0.001
Hypertension, n (%)			0.669			0.228
No	125 (47.0)	82 (49.1)		169 (46.3)	149 (51.0)	
Yes	141 (53.0)	85 (50.9)		196 (53.7)	143 (49.0)	
DM meds, n (%)			<0.001			<0.001
No	113 (42.5)	15 (9.0)		152 (41.6)	27 (9.2)	
Yes	153 (57.5)	152 (91.0)		213 (58.4)	265 (90.8)	
HTN meds, n (%)			0.587			0.623
No	170 (63.9)	111 (66.5)		232 (63.6)	191 (65.4)	

Yes	96 (36.1)	56 (33.5)		133 (36.4)	101 (34.6)	
Menopause, n (%)	–	–	–			<0.001
No	–	–		42 (11.5)	62 (21.2)	
Yes	–	–		323 (88.5)	230 (78.8)	

[1]The "Other" category included all participants who were not farmers or retirees, encompassing both employed individuals across various sectors and those unemployed.

Abbreviations: NDR, non-diabetic retinopathy; DR, diabetic retinopathy; BMI, Body Mass Index; DBP, Diastolic blood pressure; SBP, Systolic pressure; AlbF, Fructosamine/Albumin; HDL-C, High density lipoprotein cholesterol; LDL-C, Low-Density Lipoprotein Cholesterol; eGFR, estimated glomerular filtration rate; DM, diabetes mellitus; DM meds/HTN meds, use of glucose-lowering or antihypertensive medication.

Table S3. Tertile analysis of AlbF with DR in men and women (Model 5)

Sex	Tertile	AlbF range (μmol/g)	n (events)	Adjusted OR (95% CI)	P value	P for trend
Men (n=433)	T1 (lowest)	<44.59	144 (39)	1 (ref)	–	<0.001
	T2	44.59-55.44	144 (56)	1.77 (0.94–3.31)	0.076	
	T3	≥ 55.44	145 (72)	3.32 (1.61–6.87)	0.001	
Women (n=657)	T1 (lowest)	<44.59	219 (50)	1 (ref)	–	<0.001
	T2	44.59-55.44	219 (112)	3.48 (2.05–5.91)	<0.001	
	T3	≥ 55.44	219 (130)	4.30 (2.26–8.16)	<0.001	

Model 5 adjusted for age, BMI, diabetes duration, SBP, HbA1c, HDL-C, antidiabetic and antihypertensive medications; plus smoking in men only.

Table S4. Stepwise logistic regression results for AlbF (continuous, per 10 μmol/g) and diabetic retinopathy in men and women

Sex	Model	Adjustment	OR (95% CI) for AlbF (continuous)	P value
Men (n=433)	Model 1	None	1.39 (1.17–1.64)	<0.001
	Model 2	+ age, BMI	1.38 (1.16–1.65)	<0.001
	Model 3	+ DM duration, SBP, HbA1c	1.67 (1.29–2.16)	<0.001
	Model 4	+ HDL-C, smoke	1.70 (1.31–2.19)	<0.001
	Model 5	+ antidiabetic & antihypertensive medications	1.83 (1.39–2.41)	<0.001
Women (n=657)	Model 1	None	1.57 (1.38–1.80)	<0.001
	Model 2	+ age, BMI	1.47 (1.27–1.70)	<0.001
	Model 3	+ DM duration, SBP, HbA1c	1.57 (1.26–1.96)	<0.001
	Model 4	+ HDL-C	1.60 (1.28–2.00)	<0.001
	Model 5	+ antidiabetic & antihypertensive medications	1.58 (1.25–1.98)	<0.001

Model 4 for women did not include smoking because no women in the cohort were smokers. eGFR was not included in Models 1–5; it was additionally adjusted in a separate sensitivity analysis (Table 4).

Table S5. Association between AlbF (per 10 μmol/g) and diabetic retinopathy in women, stratified by menopausal status

Menopausal status	n	DR event %	OR 95CI*	P value	P for interaction
Premenopausal	104	62 (59.6)	1.87 (1.08~3.23)	0.025	0.931
Postmenopausal	553	230 (41.6)	1.52 (1.17~1.98)	0.002	

*Adjusted for age, BMI, diabetes duration, SBP, HbA1c, HDL-C, antidiabetic and antihypertensive medications.

Table S6. Association between AlbF and diabetic retinopathy in women using complete-case data without imputation (n = 517, Model 5)

Variable	n.total	n.event (%)	Model 1		Model 5	
			OR (95%CI)	P value	OR (95%CI)	P value
AlbF, per 10 μmol/g	517	219 (42.4)	1.53 (1.31~1.79)	<0.001	1.72 (1.3~2.28)	<0.001
AlbF, by tertile						
T1 (Lowest)	172	37 (21.5)	1(Ref)		1(Ref)	
T2 (Middle)	172	81 (47.1)	3.25 (2.03~5.2)	<0.001	3.39 (1.86~6.19)	<0.001
T3 (Highest)	173	101 (58.4)	5.12 (3.19~8.21)	<0.001	6.39 (3.04~13.45)	<0.001
p for trend				<0.001		<0.001

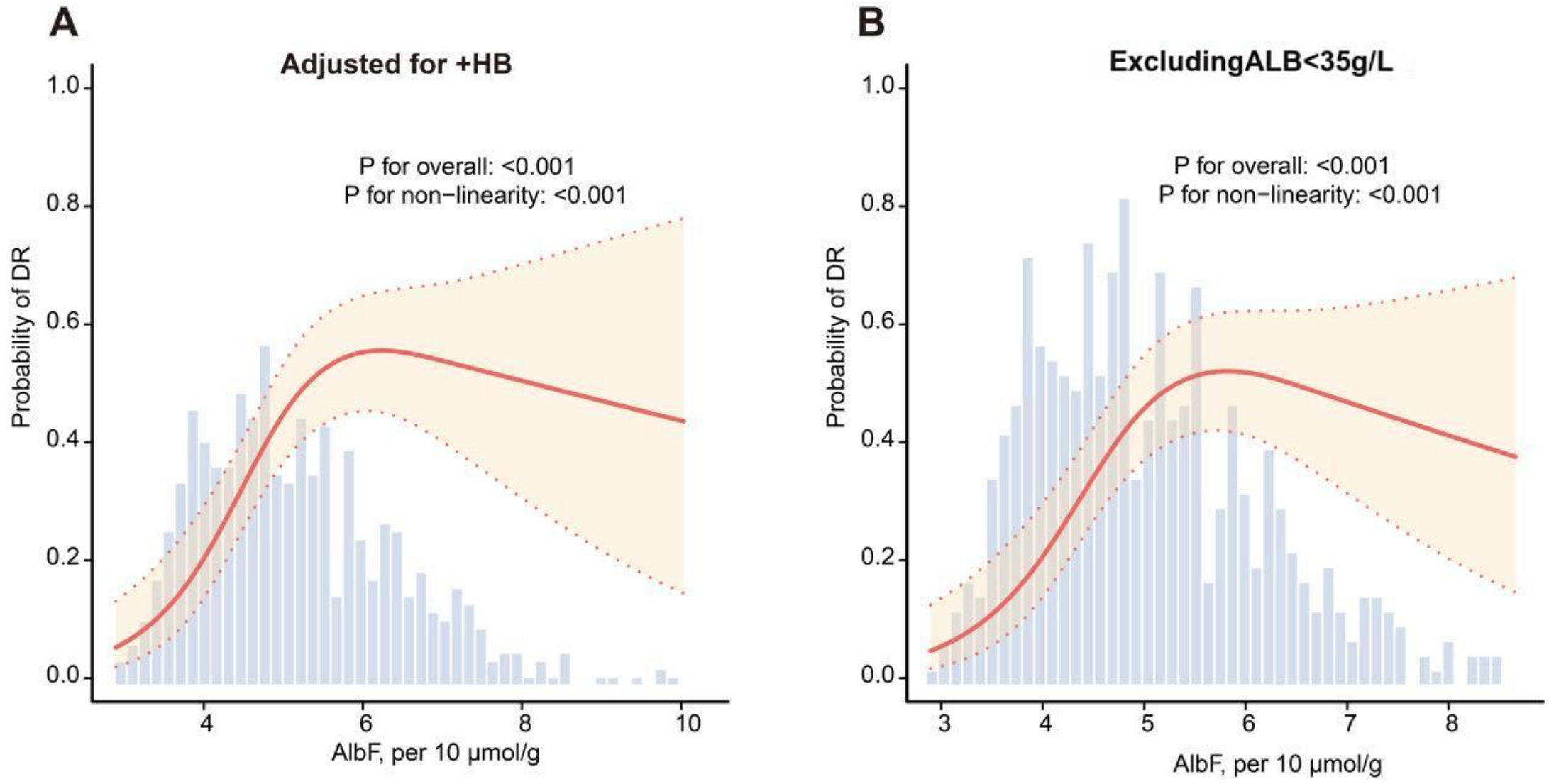

Figure S1. Sensitivity analysis of the threshold effect of AlbF on DR in women (RCS curves)

(A) Restricted cubic spline curve for the AlbF–DR association after additional adjustment for hemoglobin. (B) Restricted cubic spline curve after excluding women with serum albumin < 35 g/L (n = 585). Both models were adjusted for age, body mass index, diabetes duration, systolic blood pressure, HbA1c, high-density lipoprotein cholesterol, and use of antidiabetic and antihypertensive medications (Model 5). Shaded areas represent 95% confidence bands.

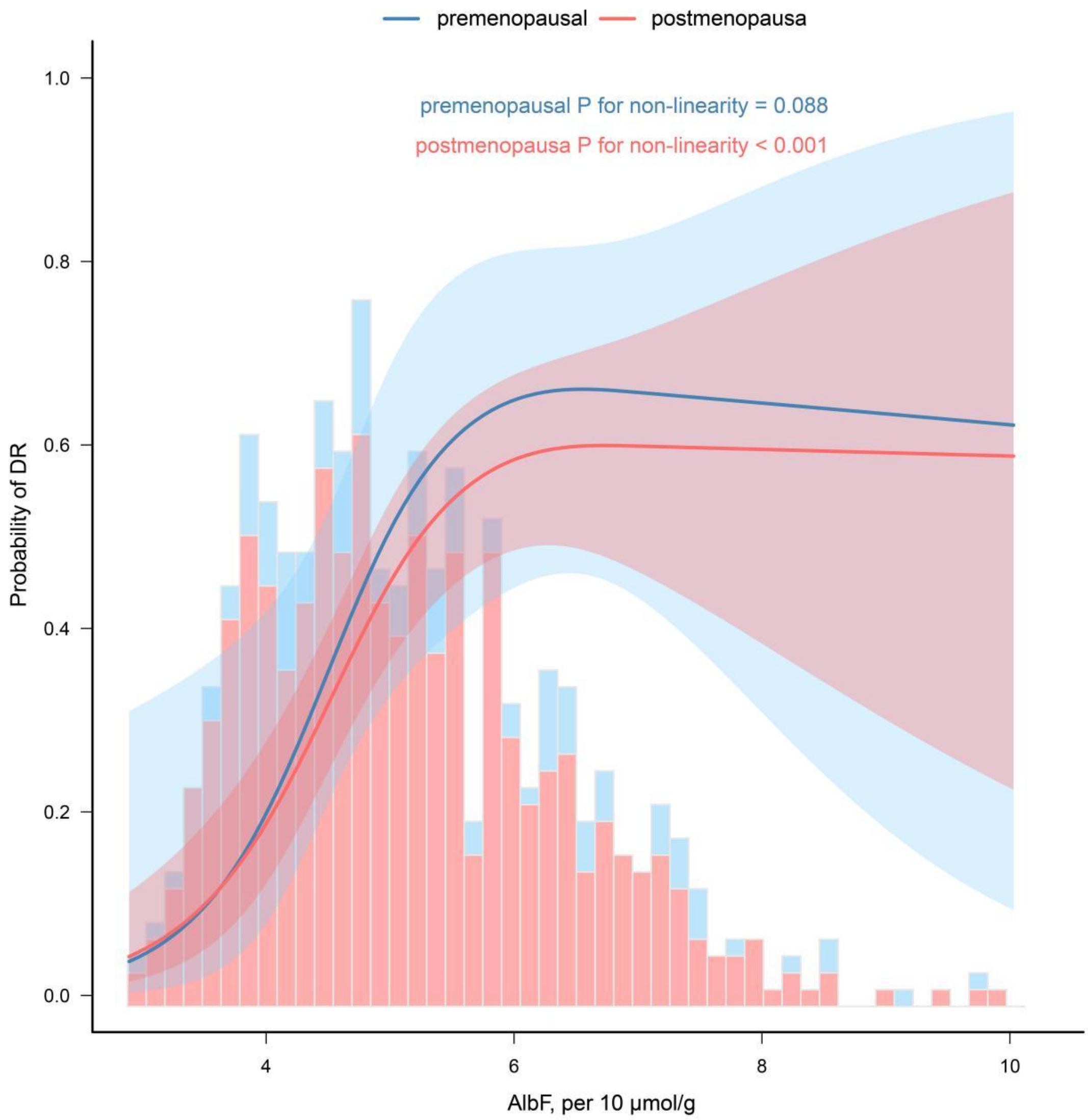

Figure S2. Restricted cubic spline curves for the AlbF–DR association in premenopausal women (n = 104) and postmenopausal women (n = 553).

Both models were adjusted for age, body mass index, diabetes duration, systolic blood pressure, HbA1c, high-density lipoprotein cholesterol, and use of antidiabetic and antihypertensive medications (Model 5). Shaded areas represent 95% confidence bands. The nonlinear threshold pattern was statistically significant in postmenopausal women ($P < 0.001$) but not in premenopausal women (P for nonlinearity = 0.088), consistent with the smaller sample size in this subgroup.
